# Animal-assisted interventions for military families: a systematic review

**DOI:** 10.3389/fpubh.2024.1372189

**Published:** 2024-05-14

**Authors:** Leanne O. Nieforth, Sarah C. Leighton

**Affiliations:** ^1^Department of Comparative Pathobiology, Purdue University, West Lafayette, IN, United States; ^2^Department of Psychology, College of Science, University of Arizona, Tucson, AZ, United States; ^3^College of Veterinary Medicine, University of Arizona, Tucson, AZ, United States

**Keywords:** family-focused interventions, assistance animals, equine-assisted, service dogs, posttraumatic stress disorder (PTSD), resilience

## Abstract

**Introduction:**

The incorporation of animals into interventions focused on military families is a relatively new concept. Though animal-assisted interventions (AAIs) have been studied in the context of military veterans, few studies incorporate members of the military family or focus on the family members’ experiences.

**Methods:**

This systematic review investigates the effects of AAIs on the wellbeing of military family members beyond the veteran themselves through three aims: (1) by describing the characteristics of AAIs for military family members, (2) by evaluating the quality of the methodology present within the current literature, and (3) by identifying key concepts and knowledge gaps within the findings reported to date.

**Results:**

A total of nine articles met the criteria to be included in the review. Though the inclusion criteria and search terms included all types of animal-assisted interventions, the only interventions represented were service dogs (*n* = 4) and equine-assisted services (*n* = 5).

**Discussion:**

Findings suggest AAIs could be beneficial in areas such as communication, relational bonds, and psychosocial well-being. Though additional research is necessary, AAIs may be an effective complementary intervention for military families.

## Introduction

Military families—a service member or veteran of the armed forces and their immediate family members—may be at risk for a multitude of concerns both inter- and intra-personally. The training and subsequent military service of the service member or veteran (hereafter, veteran) may each lead to significant emotional and mental health changes and challenges for both the veteran and their families (1, 2). As a result, family-focused interventions may be particularly effective in promoting mental and physical wellbeing and resilience in the context of military families (3). One category of complementary intervention that shows promise for military families is animal-assisted interventions (AAIs).

AAIs are defined as “any intervention that intentionally includes or incorporates animals as part of a therapeutic or ameliorative process” (4, p. 25). According to prior research, the human-animal bond that may develop during AAIs shares similarities with attachment bonds, potentially contributing to the effectiveness of these interventions (5, 6). This bond may serve as a crucial component of the therapeutic process (7). While a wide variety of literature has explored the impact of AAI for veterans [see recent reviews; (8–10)], much of the literature that has included measures of family-wide impacts has done so solely from the perspective of the veteran. The veteran’s experience may or may not align with the experiences of other family members; therefore, there is a need for studies that are designed and powered to collect data *directly* from military family members other than (or in addition to) the veterans themselves.

### Experiences of military spouses and partners

Within a close intimate relationship, the experiences of one person can also impact the other person. This is particularly true for spouses or partners of veterans due to some of the unique aspects of military service (11, 12). These unique aspects can include any combination of the veteran’s military training, deployment, combat exposure, and transition back into civilian society. During a veteran’s deployment and reintegration period, spouses and partners (hereafter, spouses) of veterans may experience psychological, logistical, and economic challenges (13, 14). Psychological challenges may include (1) supporting their veteran spouse through new physical and mental health challenges and (2) potentially also managing their own depression, anxiety, sleep disturbances, secondary posttraumatic stress, and/or suicidal ideation (15–19). These challenges may be of greater concern for spouses of veterans as deployment length increases (19). Additionally, due to the veteran’s periods of absence for military activities and forced relocation for different assignments, spouses of veterans commonly experience isolation. Frequent relocation can also create difficulties for career and identity development (12, 16). Periods of transition back to civilian life can be particularly challenging due to the change in communities and separation from the support of other military families on base (12, 16). Community social support has been shown to be an important protective factor among military families, and spouses can benefit from support to help develop new coping skills and behavior patterns (12, 20).

### Experiences of military children

Recent literature has identified a great need to recognize the experience of military-connected children and provide added support to promote their wellbeing (21). While experiences of military-connected children vary, they may include both positive and negative aspects. For example, some military-connected children experience challenges with social, behavioral, and mental health (3). If their parent’s military service takes place during childhood, separation from the parent (due to trainings or deployment) and frequent relocations have been shown to lead to decreased school performance, decreased mental health and decreased social supports (2). If a parent has been injured in combat, children may also have increased psychological concerns, with a higher incidence of mental health visits, injuries, and maltreatment (22, 23). A systematic review of the influence of a parent with war-related posttraumatic stress disorder (PTSD) on children around the world suggests that as a result of the parent’s PTSD, children may experience secondary traumatic stress, other mental health concerns, and relationship issues as adults (24).

### Intrafamilial relationships

Throughout the entire military experience from pre-deployment to reintegration, family functioning and relationships are critical to family wellbeing and resilience (25). Family functioning can also be negatively affected by the veteran’s separation from family for extended periods of time leading to a broader lack of connection (25). Furthermore, such a lack of connection may have negative effects on marital quality, relationship satisfaction, and the mental health of all military family members (26). Communication patterns, relationship quality, and parenting may also be impacted by the psychological distress of the veteran (12, 27, 28). On the other hand, effective and consistent communication among military families may be a protective factor for reducing deployment-related stressors (29). Experiences of economic strain may also impact the functioning of military families [e.g., (13, 30)].

Each family is unique, and the specifics of military culture and service may vary. However, overall, military families experience unique stressors and opportunities for growth compared to non-military families. Military families may face significant challenges resulting from military culture and military-related activities and, in the face of these challenges, many military families demonstrate significant resilience, effective coping skills, and posttraumatic growth (14). It is critical to recognize that military families experience both negative and positive effects resulting from training, deployment and reintegration, and therefore need targeted supports to bolster their wellbeing (14). It is well known that throughout their military involvement, veteran and family members’ experiences are directly impacted by social support and positive family relationships (2, 31). Family-focused interventions may be particularly effective given that they emphasize family relationships and social support strategies such as effective communication, awareness, understanding, and psychoeducation (32). Notably, it has been suggested that social support theory may be a key mechanism through which AAIs promote change, indicating strong potential for their effectiveness for military families (33).

To synthesize the existing literature related to the influence of AAIs on military families, we conducted a systematic review. Given the potential discrepancies between the veterans’ own experience and that of their family members, the present review was focused solely on studies which collected data *directly* from military family members other than (or in addition to) the veterans themselves. The research question was “What are the effects of AAIs on wellbeing of military family members?” Our first aim was to describe the characteristics of AAIs for military family members. Our second aim was to evaluate the quality of the methodology present within the current literature. Our third and final aim was to identify key concepts and knowledge gaps within the findings reported to date.

## Methods

We conducted a systematic literature review in accordance with PRISMA guidelines. A total of five databases were searched from their inception date to 1 March 2023 (ERIC, ProQuest Research Library, ProQuest Dissertations and Theses, PsycINFO, PubMed, and Scopus). In addition, the Human-Animal Bond Research Institute (HABRI) Central Database and the Journal of Veteran Studies were hand-searched for relevant articles. Search terms included a term for military families and a term for animal-assisted or pet. The terms for military families included: military and/or veteran families/spouses/partners/children/couples. The terms for animal-assisted/pet were adapted from a previous AAI literature review (34), and included: animal intervention, animal therapy, animal assisted, animal facilitated, anthrozoology, assistance animal(s), assistance dog(s), assistance horse(s), canine therapy, canine assisted, canine facilitated, companion animal(s), dog therapy, dog assisted, dog facilitated, dolphin therapy, dolphin assisted, dolphin facilitated, equine therapy, equine assisted, equine facilitated, hippotherapy, horseback riding, human animal bond, human animal interaction(s), pet therapy, pet assisted, pet facilitated, service animal(s), service dog(s), service horse(s), therapeutic animal(s), therapeutic dog(s), therapeutic horse(s), therapeutic horseback, therapeutic pet(s), therapeutic riding, and therapy with animals. Specific search formatting was adapted according to the syntax rules for each database. While this review was not pre-registered, the protocol was developed in advance, prior to conducting the database search.

Inclusion criteria for articles included:

1. Publication in English in a peer-reviewed journal or as a dissertation
2. Collection of empirical data on AAIs
3. Reporting of outcome results for military families, directly from family members other than the veteran

Exclusion criteria included:

1. Not published in English
2. Literature reviews/meta-analyses
3. Validation of a measure
4. Book review or book chapter
5. Magazine article, commentary, or editorial without empirical outcomes
6. Conference presentation, abstracts, or posters
7. Reporting family outcomes only from the perspective of the veteran (i.e., no collection of data directly from other family members)

**Figure 1:**
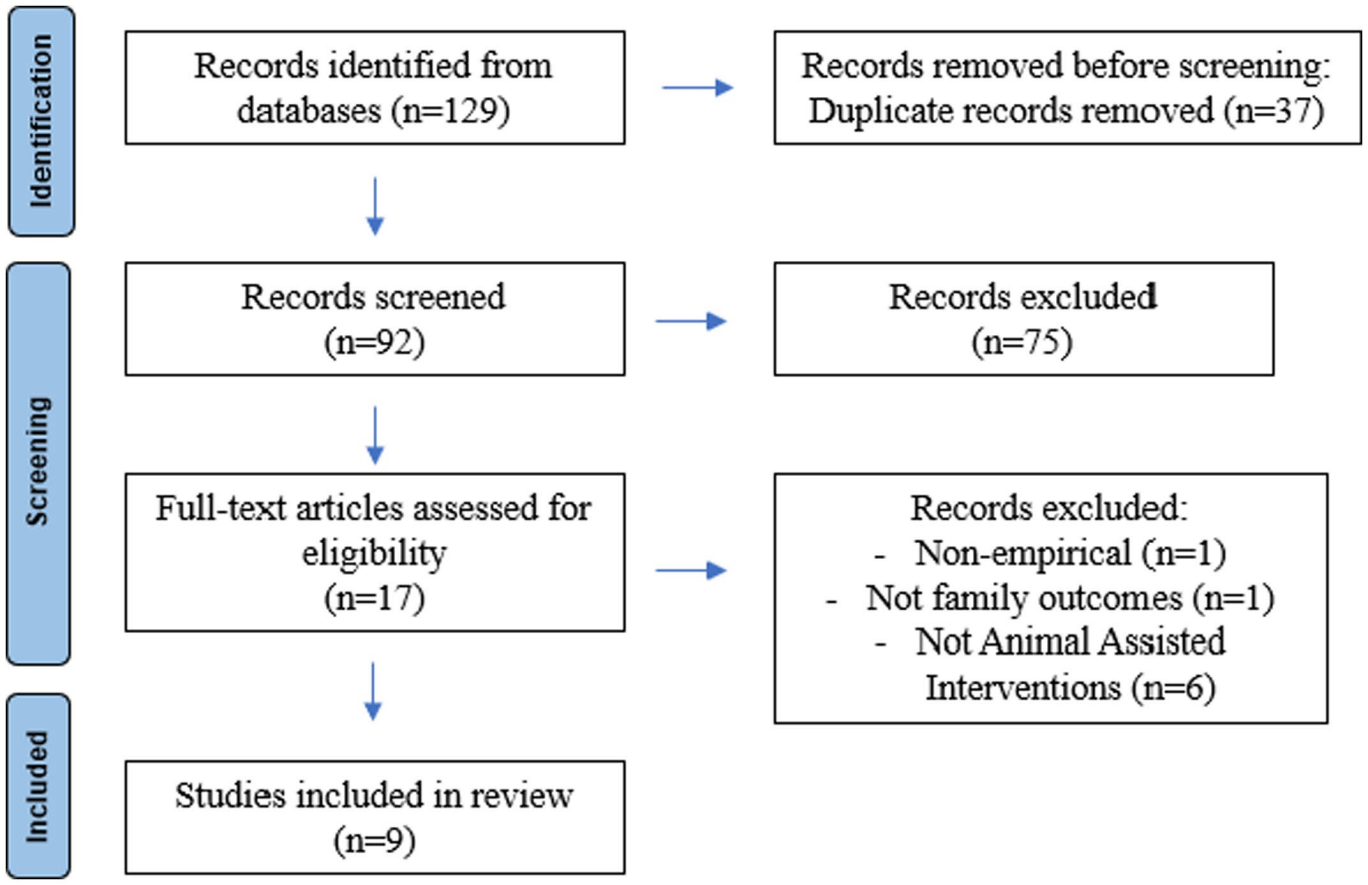
PRISMA flow diagram.

Both authors conducted an initial screening for inclusion based on titles and abstracts. Selected articles were then read in full by both authors to make a final determination (Cohen’s Kappa = 1.0). Disagreements were resolved through discussion. Article screening flow is presented in Figure 1. Both authors independently coded 20% of the articles to establish adequate inter-rater reliability. Finally, the first author (LN) coded 100% of the articles.

Methodological rigor was assessed adapting binary (yes/no) scoring questions from a previous human-animal interaction review (see Supplementary Material) (9). Questions focused on transparency of study methods (e.g., Was the aim of the study stated? Was there a clear description of participant eligibility? Was ethical approval attained?); the study design (quantitative, qualitative, or mixed methods) dictated the applicable methodology-specific questions.

## Results

To date, nine articles have been published that specifically collected data focused on AAIs *directly* from military family members (Table 1). Four articles (44%) focused on the influence of equine-assisted services (EAS) for military families and five (56%) focused on the influence of service dogs on military families. Three of the five articles focused on service dogs were from the same clinical trial, each reporting on a different data stream of the parent trial. None of the articles that met our eligibility criteria for inclusion included AAIs with animals other than horses or dogs. One of the articles (11%) was an unpublished dissertation. Eight of the nine articles (89%) reported findings from studies that were completed in the United States, and one that was completed in Germany (11%). The articles were published in a range of interdisciplinary journals. The earliest article was published in 2018 and the most recent in 2022 with seven of the nine articles (78%) published within 3 years prior to the search being conducted.

**Table 1:** Included article characteristics.

Article Title	Authors	Year	Country	Journal	AAI
Evaluation of an equine-assisted therapy program for veterans who identify as “wounded, injured or ill” and their partners	Romaniuk, Evans, and Kidd	2018	USA	PLoS ONE	Equine-assisted services
The effects of gestalt-centered equine facilitated therapy on marital satisfaction in relationships in which one member is a combat veteran suffering with post traumatic stress disorder	Skidmore	2018	USA	ProQuest Dissertations and Theses	Equine-assisted services
Acceptability of an adjunct equine-assisted activities and therapies program for veterans with posttraumatic stress disorder and/or traumatic brain injury	Sylvia, West, Blackburn, Gupta, Bui, Mahoney, Duncan, Wright, Lejeune, and Spencer	2020	USA	Journal of Integrative Medicine	Equine-assisted services
“A part of our family”? effects of psychiatric service dogs on quality of life and relationship functioning in military-connected couples	McCall, Rodriguez, MacDermid Wadsworth, Meis, and O’Haire	2020	USA	Military Behavioral Health	Service dogs
Understanding partner perceptions of a service dog training program for veterans with PTSD: building a bridge to trauma resiliency	Whitworth, O’Brien, Wharton, and Scotland-Coogan	2020	USA	Social Work in Mental Health	Service dogs
Equine-assisted psychotherapy with traumatized couples‚ improvement of relationship quality and psychological symptoms	Willmund, Zimmerman, Alliger-Horn, Varn, Fischer, Parent, Sobottka, Bering, Rose, Ströhle, and Köhler	2021	Germany	Journal of Marital and Family Therapy	Equine-assisted services
PTSD service dogs foster resilience among veterans and military families	Nieforth, Craig, Behmer, MacDermid Wadsworth, and O’Haire	2023	USA	Current Psychology	Service dogs
Posttraumatic stress disorder service dogs and the wellbeing of veteran families	Nieforth, Miller, MacDermid Wadsworth, and O’Haire	2022a	USA	European Journal of Psychotrau matology	Service dogs
Quantifying the emotional experiences of partners of veterans with PTSD service dogs using ecological momentary assessment	Nieforth, Abdul Wahab, Sabbaghi, MacDermid Wadsworth, Foti, and O’Haire	2022b	USA	Complementary Therapies in Clinical Practice	Service dogs

Listed order corresponds to publication date (oldest to most recent).

### Aim 1: Characteristics of AAIs for military family members

The nine included articles (4 focused on EAS, 5 on service dogs) represent seven different studies focused on military families and AAIs (Table 2). Three out of the four EAS studies (75%) took place at private facilities. The fourth study took place at a Professional Association of Therapeutic Horsemanship (PATH) accredited facility. Activities within the EAS programs covered a broad range, but most were non-riding related programming (75% of studies). Program duration ranged from 2 days to 5 weeks and the total contact hours were unclear.

**Table 2:** Aim 1: characteristics of AAI for military families.

Study	AAI terminology	Species	Setting	Program Activities	Program Duration
Romaniuk et al. (35)	Equine-assisted	Equine	Private horse farm	Relational Gestalt therapy and groundwork with horses	5 days (hours not specified)
Skidmore (36)	Equine-facilitated	Equine	PATH accredited facility	Grooming, obstacle courses, joining up/haltering horses	5 weeks for 1 h/week
Sylvia et al. (37)	Equine-assisted	Equine	Private horse farm	Therapeutic riding and driving, equine-assisted learning (EAL)/groundwork, horse-human energy work, herd observation, equine care + non horse activities (quilting)	2 days (three 2-h sessions per day)
McCall et al. (38)	Service dogs	Canine	Family home	-	3-week placement class
Whitworth et al. (39)	Service dogs	Canine	Family home	-	Not specified
Willmund et al. (40)	Equine-assisted	Equine	Private horse farm	Observe horses, meet horses, “From Here to There” (build and create their life path utilizing obstacles), take a horse through obstacles, follow up activity (resource discussion, reflection)	3 days (hours not specified)
Nieforth et al. (41)	Service dogs	Canine	Family home	-	3-week placement class
Nieforth et al. (42)	Service dogs	Canine	Family home	-	3-week placement class
Nieforth et al. (43)	Service dogs	Canine	Family home	-	3-week placement class

Given the nature of service dog partnerships, in that the service dog lives alongside the military family, the setting for each study incorporating service dogs was in the family home. Aside from the initial placement program (e.g., 3-weeks intensive pairing and training process at the service dog provider’s facility), there were no planned program activities nor reported duration for the service dog interventions. This is expected given the nature of the service dog intervention; the service dogs are trained in specific tasks to mitigate the handler’s symptoms and fully integrated into the handler’s daily life until the time of the service dog’s retirement or death.

### Aim 2: Quality of methodology

The sample size of articles ranged from 15 to 120 participants (*M* = 71.11, *SD* = 40.73). Five articles (56%) incorporated both the veterans and the spouses, and four articles (44%) only included spouse participants. No articles collected data directly from military family children. The average age across all articles that reported participant age was approximately 39.64 years old (SD = 5.01). Excluding Sylvia et al. (37) (where participants could have been counted twice if both a veteran and a spouse), across all articles, there were approximately 330 individuals who identified as female (58%) and 204 who identified as male (42%). Only four articles (44%) reported on race and/or ethnicity. Six articles (67%) were longitudinal and used survey measures. Three articles (33%) were cross-sectional and used survey measures. One of the cross-sectional articles (11%) also incorporated qualitative interviews. All the articles that methodologically could have reported an effect size did so. Two articles (22%) reported results from studies using within-subject designs, three articles (33%) reported results from studies employing a waitlist control group and one article (11%) reported results from a study employing a non-intervention control group (Table 3).

**Table 3:** Aim 2: methodology.

Study	Sample size	Age (*M*)	Gender	Race/Ethnicity reported	Design	Effect size	Comparison condition
Romaniuk et al. (35)	25 SM	50.28 individ.	SM: 29 M, 10 F	N	Within subject	*d*	Within subjects
	22 spouses	40.82 couples	Spouses: 1 M, 7 F				
Skidmore (36)	2 couples	34.75	SM: 2 M	Y	Single-subject	-	Within subjects
			Spouses: 2 F				
Sylvia et al. (37)	62 SM	37.95 SM	SM: 54 M, 11 F^a^	Y	Mixed methods (Qualitative + Cross-sectional)	-	NA
	44 family	39.42 family	Family: 11 M, 38 F^a^				
McCall et al. (38)	60 SM-spouse dyads	36.68 SM	SM: 51 M, 9 F	N	Mixed methods (Qualitative + Cross-sectional)	*g*	Waitlist
		NS—spouses	Spouses: 7 M, 53 F				
Whitworth et al. (39)	15 spouses	49 spouses	Spouses: 2 M, 13 F	N	Qualitative	-	-
Willmund et al. (40)	36 couples	40.35 SM	SM: 34 M, 2 F	N	Non-randomized control	*d*	Non-intervention control
		38.63 spouses	Spouses: 2 M, 34 F				
Nieforth et al. (41)	67 SM	NS	SM: 53 M, 14 F, Spouses: 1 M, 33 F	N	Qualitative	-	-
	34 spouses						
Nieforth et al. (42)	88 spouses	36.5 spouses	Spouses: 10 M, 78 F	Y	Non-randomized control	*d*	Waitlist
Nieforth et al. (43)	87 spouses	36.5 spouses	Spouses: 10 M, 77 F	Y	Non-randomized control	*d*	Waitlist

SM, service members or veterans; NS, not specified; *d*, Cohen’s *d*; *g*, Hedge’s *g*. ^a^These numbers may not correspond because some of the spouses were included in the service member demographics, because they were also service members themselves.

**Figure 2:**
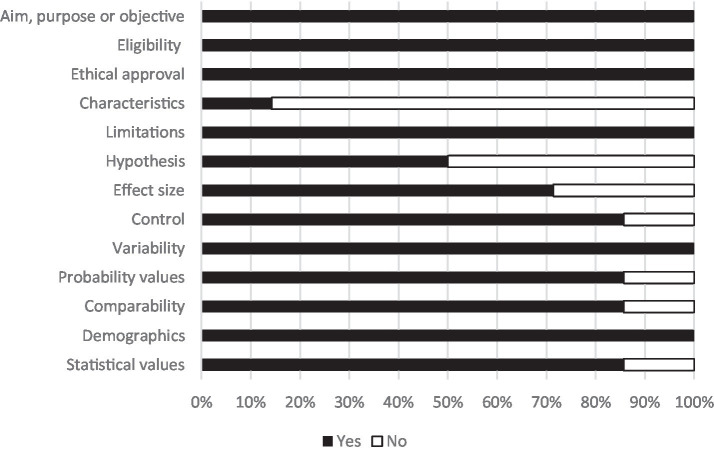
Methodological rigor: articles quantitative components (*n* = 7).

**Figure 3:**
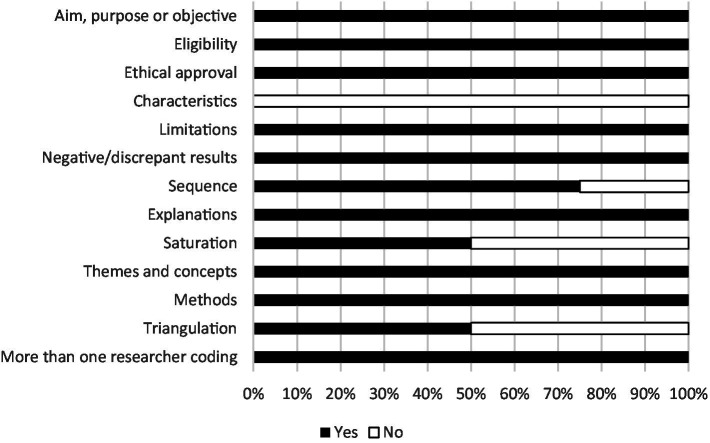
Methodological rigor: articles with qualitative components (*n* = 4).

Analysis of methodological rigor identified strengths of the current literature as well as areas for growth. All quantitative studies stated variability, limitations, demographics, ethical approvals, aims and eligibility. Six out of seven quantitative studies stated comparability of baseline characteristics, statistical values, probability values and the control group. Five out of seven mentioned effect sizes and fewer than half of studies explicitly stated a hypothesis or characteristics of the animals incorporated into the intervention (Figure 2). All qualitative studies stated aims, eligibility, ethical approval, coherent explanations of findings, clear methodology, limitations, clear themes, incorporated more than one researcher into coding and considered negative/discrepant results. Three out of four incorporated sequences from the original data set. Half of qualitative studies discussed achieving saturation and the triangulation of data. No qualitative studies mentioned the characteristics of the animals incorporated into the intervention (Figure 3).

### Aim 3: Key concepts reported to date

Across manuscripts there were a wide variety of measures used to assess military family wellbeing. Based on findings reported in the articles, negative impacts from the AAIs were minimal. The most commonly used measure was the PTSD Checklist which was incorporated in four articles (44%) (35, 38–40). The other measures that were repeated (twice) included the Patient Health Questionnaire, Patient-Reported Outcomes Measurement Information System (PROMIS) Anxiety, PROMIS depression, PROMIS Social Isolation, PROMIS Companionship, PROMIS Ability to participate in social activities, Connor Davidson Resilience Scale, and the Bradburn Scale of Psychological Wellbeing (38, 42) (Table 4).

**Table 4:** Aim 3: key concepts.

Study	Assessment measures	Outcomes
Romaniuk et al. (35)	DASS-21	Individual program participants: Significantly lower scores on the DASS-21 and PCL-5 at posttest compared to pretest. Significantly higher scores on the OHQ and Q-LES-Q-SF at posttest compared to pretest. Significantly higher scores on the DASS-21 and the PCL-5 at follow up. Significantly lower scores on the OHQ and Q-LES-Q-SF at follow up. No significant differences between pre and follow up on any measures. Benefits of the individual program short term (not retained at follow up). Couples program participants: Significantly lower scores on depression and stress (DASS 21) and PCL 5 at posttest and follow up compared to pretest. Fewer participants in the couples program meet criteria for PTSD at follow up. No significant difference in anxiety (DASS 21) pretest to posttest. No significant difference in DASS 21 and PCL between posttest and follow up. Significant reductions in depression, stress and PTSD that remained at follow up.
	PCL-5	
	OHQ	
	Q-LES-Q-SF	
Skidmore (36)	DAS	Intervention has social validity. Participating SMs had a small treatment effect. Participating spouses had a medium to large treatment effect.
Sylvia et al. (37)	Satisfaction surveys	Qualitative themes: Horses can catalyze emotional rehabilitation. Effectiveness of immersion in equine-assisted activities. Healing through mindfulness and relaxation. Necessity of education about PTSD from staff. Overall program experience: SM rate the program 9.76/10. Spouses rate the program 9.91/10. Program Pros: disconnection from electronic devices, being around animals, knowledgeable staff. Cons: would prefer better communication about the program, want the program to be longer and potential additional activities. Parts of the program that were working well: rehabilitation, logistical aspects of program, quality of info provided, and overall experience.
McCall et al. (38)	PROMIS Anger, Anxiety, Depression, Social Isolation, Companionship, Ability to Participate in Social Activities	No significant differences, but meaningful effect sizes apparent. Qualitative data suggests substantial and positive benefits for spouses which are not apparent in the quantitative data. Spouses report lower levels of anger, higher levels of resilience, less social isolation, greater companionship, less health-related impairment at work, and greater relationship satisfaction. SM report fewer problems with family functioning and affective responsiveness, greater relationship satisfaction. 81% of codes described benefits of service dogs. 17% of qualitative codes described challenges of service dogs.
	PHQ	
	VR-12	
	BSPW	
	SLS	
	CDRS	
	WPAIQ	
	MFAD	
	RAS	
	PCL-4	
	Qualitative Prompts	
Whitworth et al. (39)	PCL-5	Development of a conceptual model based upon the understanding of how spouses believe the program helps the SM, the spouses themselves and their relationships. They experience challenges but form a relational bridge (bond with SD, support in program, close spouse connection) that promotes resiliency. Spouses are symptomatic for PTSD (73% had a total severity score > 35). Generally high levels of relationship satisfaction
	RAS	
	Qualitative interviews	
Willmund et al. (40)	LEC	SM Significant improvement in PCL-5 negative alterations in cognition and mood scale. Significant improvement in total severity score for PCL-5. PL (Problem List) score significantly improved. Decrease in PHQ-9 depression.Spouses Significant improvement for spouse in PFB scales (commonality/communication, global happiness), total PL score, and # of difficult problem areas/severity of somatic symptoms (PHQ-15).
	PCL-5	
	PHQ-15	
	Partnership Questionnaire	
	PL	
Nieforth et al. (41)	Qualitative open-ended survey	Themes: Service dogs help to build emotional reserves. Service dogs increase relational load. Service dogs facilitate relational maintenance behaviors leading to communal orientation.
Nieforth et al. (42)	BSPW	Significant increase in number of activities among spouses of SM with service dogs. Significant increase in caregiver burden among spouses of SM with service dogs. Significant decrease in caregiver satisfaction among spouses of SM with service dogs. Non-significant findings: all child outcomes and all outcomes not listed above.
	CDRS	
	AQ	
	RCAS	
	ZCB	
	PROMIS Anxiety, Depression, Companionship, Ability to Participate in Activities, Social Isolation, Pediatric Positive Affect, Pediatric Psychological Stress	
	MDORS	
	IOS	
	LAPS	
	PQLI-FI	
Nieforth et al. (43)	Modified version of PANAS and DEQ	Higher levels of positive emotion (calmness and confidence) in the service dog group compared to waitlist group. No significant differences for negative emotions. No significant differences regarding social spouse proximity.
	Binary Social Partner Proximity question	

SM, service members or veterans; DASS-21, Depression Anxiety Stress Scale-21; PCL, PTSD Checklist; OHQ, Oxford Happiness Questionnaire; Q-LES-Q-SF, Quality of Life Enjoyment and Satisfaction Questionnaire-SF; DAS, Dyadic Adjustment Scale; PROMIS, Patient Reported Outcomes Measurement Information System; PHQ, Patient Health Questionnaire; VR-12, Veterans Rand 12-item Health survey; BSPW, Bradburn Scale of Psychological Wellbeing; SLS, Satisfaction with Life Scale; CDRS, Connor Davidson Resilience Scale; WPAIQ, Work Productive and Activity Impairment Questionnaire; MFAD, McMaster Family Assessment Device; RAS, Relationship Assessment Scale; LEC, Life Events Checklist; PL, Problem List; AQ, Activity Questionnaire; RCAS, Revised Caregiver Appraisal Scale; ZCB, Zarit Caregiver Burden; MDORS, Monash Dog-Owner Relationship Scale; IOS, Inclusion of Self in Others Scale; LAPS, Lexington Attachment to Pets; PANAS, Positive and Negative Affect Scale; DEQ, Discrete Emotions Questionnaire.

#### Equine-assisted services and military families

Results suggest that equine-assisted services (EAS) may be beneficial to military families; however, the number of articles is limited, and further research is needed. The current literature suggests improvements to interpersonal relationships such as reduced relationship problems, improved relationship quality and improved communication (40). Additionally, the literature suggests potential mental health benefits including reduction of depressive symptoms, somatic symptoms, and PTSD symptoms (35, 40). Qualitative findings suggest horses may act as a catalyst for emotional rehabilitation and healing and suggest social validity of the intervention (36, 37). There were no negative findings presented in the EAS literature on military families.

#### Service dogs and military families

Results suggest that service dogs may also be beneficial to military families. Quantitatively, the presence of a service dog may increase the number of activities spouses are involved in and promote a higher amount of positive emotions (42, 43). Qualitatively, service dogs may promote resilience in military families (39, 41). Two mechanisms for this resilience process have been suggested. First, one study suggests that a conceptual model of resilience emerges as veterans build a three-part “relational bridge” made up of reduction of PTSD symptoms, increased resilience and improved relationship functioning through the service dog intervention between the veteran and their community (39). This “bridge” helps to promote resilience in navigating challenges both as individuals and couples (39). Second, service dogs may promote a sense of communal orientation—a sense of “we-ness”—within the couple, by facilitating both positive and negative interactions (41). Additionally, service dogs may have overarching positive benefits for mental well-being and quality of life to both the veteran and the spouse (38). Aside from benefits of service dogs to military families, the quantitative data suggests a few challenges that do not appear to be captured by qualitative findings to date (38). One article found that spouses of veterans with service dogs may experience increased caregiver burden and decreased caregiver satisfaction in comparison to spouses without service dogs in their homes (42). In addition to the benefits and challenges mentioned above, it is important to note that many findings across articles were not statistically significant, but in some cases had meaningful effect sizes (38).

## Discussion

The overarching research question guiding this review was, “What are the effects of AAIs on wellbeing of military family members?” A total of nine articles met the criteria to be included. Overall, findings support the notion that AAIs may be well-suited as a complementary family-focused intervention for military families, but more research is needed.

Thus far, limited research focused on AAI for military families has examined two types of AAI: service dog placements and equine-assisted services. Across articles, the equine-assisted services varied widely from observation to groundwork to mounted riding, whereas the characteristics of the service dog placements were relatively homogeneous. This is unsurprising given that four out of the five service dog studies included dogs trained and placed by the same service dog provider. A fruitful direction for future research will be to explore other types of AAIs (beyond equine-assisted interventions and service dog partnerships) and to incorporate multiple providers.

AAIs have the potential to help not only families with ongoing mental or physical health concerns, but also families with other types of challenges such as general family functioning, stress reduction, and post-deployment readjustment. Though not studied in the context of military families, there is preliminary data on other types of AAIs that may be effective for addressing concerns relevant to military families. For example, studies suggest that participation in care farms may provide benefits to mental health (44, 45) and that therapy dog visits or the presence of facility dogs may have positive effects on wellbeing (46, 47). Though these AAIs have solely been studied for individual participants, it seems plausible that similar impacts could be found for families. Additionally, though not a goal-oriented AAI, the role of companion animals in military families should be a continued focus. Given the perception of companion animals as family members, they may have an important role in family functioning and communication for military families.

A major gap evident in the current body of literature is the lack of knowledge surrounding the bi-directional influence of one family member’s outcome on another family member’s outcome. Future studies should, for example, consider associations between family member outcomes and plan for analyses capable of identifying key predictors at both the individual *and* the dyad level. Similar methodology has, for example, been incorporated into a study of children with service dogs for autism and their families, providing a possible template for exploring the quantitative influence of human-animal interactions within family systems [e.g., (48)]. Additionally, instead of being an add-on to studies focused primarily on veterans, it is important for studies to be designed and powered to specifically study family-focused interventions, considering the military family as a whole unit.

Key concepts found in the current literature regarding AAIs for military families are aligned with the broader literature on military family-focused interventions. In both the literature on military family-focused interventions more broadly and the literature on AAIs for military families, interventions primarily take place in the context of a veteran receiving a diagnosis of PTSD, or when intimate partner violence has occurred (49). Family involvement in treatment for PTSD has been shown to potentially reduce dropout rates, which are a major concern for treatment success (50). Multiple evidence-based family-focused intervention frameworks exist for the treatment of PTSD (51). Future studies should consider combining these already evidence-based approaches with AAI, for example combining a therapy dog visit with cognitive behavioral conjoint therapy, to explore the potential cumulative effects of the interventions.

Only one study reported findings related to the experience of military children. This mirrors the general military family intervention literature, which also has limited studies on the experience of children in comparison to other family members (21). Given that children in military families can experience unique challenges and concerns, this is an important area for future exploration.

Overall, the quality of the methodology for studies in this review was average and there is room for improvement. The number of articles was limited, and there was significant variation in the measures used to measure outcomes. When studies do not consistently employ the same outcome measures, it prevents conducting a meta-analysis, which is important to establish an evidence base for any intervention or complementary intervention. Furthermore, there are currently no randomized clinical trials comparing outcomes for AAIs to other types of family-focused interventions. Without randomization, no causal claims can be made about the impacts of AAIs for military families.

Despite these gaps, preliminary evidence suggests that AAIs may be effective for military families. Outcomes from the current review were primarily positive, and challenges highlight opportunities to refine or improve the interventions themselves. Though this review was focused on studies that reported outcomes of military family members (other than or in addition to veterans), findings are also relevant to practitioners of interventions aimed at individual family members. For example, although a service dog intervention is typically targeted toward a single family member (the veteran with a disability), studies suggest that the service dog intervention may have an influence that extends to the whole family (39, 41). This is unsurprising given that family systems are complex and what happens to one family member (positive or negative) ripples through the rest of the family (52). The “ripple effect” of service dog partnerships throughout the whole family system is notable and important for providers to consider, and further underscores the possibility that family-focused interventions might be particularly suitable to military families.

### Limitations

A few limitations should be considered with the results of this systematic review. First, though we searched a variety of databases, and made every effort to capture all relevant articles, it is possible that some relevant articles were missed. Second, given the limited amount of published literature on this topic, findings should be interpreted with caution as broad claims are unable to be made.

## Conclusion

Within the military family literature there is a call to include all members of the military family in both the interventions and the research, beyond solely the veterans (12, 21, 53). AAIs are a promising potential answer to this call because they can be highly individualized and may impact the entire family. In the case of equine-assisted services, the whole family can participate together. In the case of service dog placements, the dogs often interact with all family members even though they are specifically trained for the veterans.

Military families need support to promote resilience and diminish concerns with mental or social health following military-related challenges. The mental and social health concerns that they may experience have the potential to be addressed through AAIs focused on and developed for the entire military family unit, rather than solely for individual members. Despite this significant potential, very few articles in the literature to date have focused on AAIs for military families as the primary population. Despite the limited quantity of studies, preliminary data suggests that in conjunction with other services, animal-assisted interventions may have the potential to promote resilience and wellbeing in military families.

## Data availability statement

The original contributions presented in the study are included in the article/Supplementary material, further inquiries can be directed to the corresponding author.

## Author contributions

LN: Conceptualization, Data curation, Funding acquisition, Formal analysis, Investigation, Methodology, Project administration, Supervision, Writing – original draft, Writing – review & editing. SL: Data curation, Formal analysis, Investigation, Methodology, Writing – original draft, Writing – review & editing, Funding acquisition.
