# Supplementary material for: Animal-assisted interventions for military families: a systematic review
Source: Front Public Health. 2024 May 14;12:1372189. doi: 10.3389/fpubh.2024.1372189 (PMC11135175; doi:10.3389/fpubh.2024.1372189)
Supplement: Supplementary file 1 [file Data_Sheet_1.docx]

**Appendix 1-** Methodological Rigor Scoring Questions

**Questions for All Studies**

1. Was an aim, purpose, objective, or research question of the study stated?
2. Is there a clear description of eligibility (inclusion/exclusion) criteria of participants?
3. Was ethical approval sought and received, and clearly stated including source?
4. Were characteristics of the animals in the study described, including their provider and training?
5. Were limitations of the study discussed in detail considering sources of potential bias or imprecision?

**Questions for Quantitative Study Components**

1. Was a hypothesis/hypotheses stated?
2. Are effect sizes for most outcomes provided?
3. Does the design include a control condition?
4. Does the study provide estimates of the variability in the data for most outcomes?
5. Have actual probability values been reported for most outcomes?
6. Was there a demonstration that groups or baseline characteristics were comparable on demographic and medical variables?
7. Were key demographic characteristics of study participants described including average age and percent male/female?
8. Were statistical values for most outcomes reported?

**Questions for Qualitative Study Components**

1. Are negative/discrepant results considered?
2. Are sequences from the original data presented and were these fairly selected?
3. Are the explanations for the results plausible and coherent?
4. Do the authors report achieving data saturation?
5. Is it clear how the themes and concepts were identified in the data?
6. Is it clear what methods were used to collect data with sufficient details, including type of method and tools?
7. Is there triangulation of data?
8. Was the analysis performed by more than one researcher?

Adapted from Leighton et al., 2022
